# The Modern Double-Poling Technique Is Not More Energy Efficient Than the Old-Fashioned Double-Poling Technique at a Submaximal Work Intensity

**DOI:** 10.3389/fspor.2022.850541

**Published:** 2022-05-18

**Authors:** Tomas Carlsson, Wilma Fjordell, Lars Wedholm, Mikael Swarén, Magnus Carlsson

**Affiliations:** ^1^School of Health and Welfare, Dalarna University, Falun, Sweden; ^2^Swedish Unit for Metrology in Sports, Dalarna University, Falun, Sweden

**Keywords:** cross-country skiing, gross efficiency, oxygen uptake, blood lactate concentration, biomechanical analysis, kinematics, double poling

## Abstract

The purpose of the study was to investigate whether there are energy-efficiency differences between the execution of the old-fashioned double-poling technique (DP_OLD_) and the modern double-poling technique (DP_MOD_) at a submaximal work intensity among elite male cross-country skiers. Fifteen elite male cross-country skiers completed two 4-min tests at a constant mechanical work rate (MWR) using the DP_MOD_ and DP_OLD_. During the last minute of each test, the mean oxygen uptake (VO_2_) and respiratory exchange ratio (RER) were analyzed, from which the metabolic rate (MR) and gross efficiency (GE) were calculated. In addition, the difference between pretest and posttest blood-lactate concentrations (BLa_diff_) was determined. For each technique, skiers' joint angles (i.e., heel, ankle, knee, hip, shoulder, and elbow) were analyzed at the highest and lowest positions during the double-poling cycle. Paired-samples *t*-tests were used to investigate differences between DP_MOD_ and DP_OLD_ outcomes. There were no significant differences in either VO_2_mean, MR, GE, or BLa_diff_ (all *P* > 0.05) between the DP_MOD_ and DP_OLD_ tests. DP_MOD_ execution was associated with a higher RER (*P* < 0.05). Significant technique-specific differences were found in either the highest and/or the lowest position for all six analyzed joint angles (all *P* < 0.001). Hence, despite decades of double-poling technique development, which is reflected in the significant biomechanical differences between DP_OLD_ and DP_MOD_ execution, at submaximal work intensity, the modern technique is not more energy efficient than the old-fashioned technique.

## Introduction

From a physiological perspective, endurance performance is suggested to be determined by the sum of the aerobic and anaerobic energy contributions multiplied by gross efficiency (GE) (Joyner and Coyle, 2008). In line with this model, GE has been suggested to be important for performance in elite male cross-country skiing; more specifically, skiers with higher performance levels have been found to have a higher GE than skiers with lower levels of performance (Sandbakk et al., 2010; Ainegren et al., 2013). Cross-country skiers use different sub-techniques during a competition to minimize energy expenditure and/or maximize skiing speed on course sections with different inclinations (Welde et al., 2017; Strøm Solli et al., 2020).

The four main sub-techniques for propulsion in the classical technique are the diagonal-stride technique (DS), double-poling technique (DP), double-poling technique with leg kick (DPK), and herringbone technique (Nilsson et al., 2004). In the DS, the arm's force is transferred through the pole simultaneously with the push off with the leg on the contralateral side of the body (Nilsson et al., 2004). The DP is characterized by a parallel movement of the arms with a synchronous force transfer solely through the poles. However, while using the DPK, the force contribution for propulsion comes from both the synchronous pole-force transfer used in the DP and a simultaneous kick with one of the legs, where the force is transferred to the ground by a kicking motion (Nilsson et al., 2004).

Traditionally, the predominant sub-technique in classical cross-country skiing was the DS, and this rhythmical movement was occasionally broken with the use of the DP or DPK (Saltin, 1996). During recent decades, there have been great developments related to track preparation, functional characteristics and reduction of the mass of ski equipment (Street, 1992). These developments accompanied by improved upper-body strength/power and technique development in elite skiers (Holmberg et al., 2005; Stöggl and Holmberg, 2011, 2016), have led to changes in skiers' sub-technique usage. Recently, it has been shown that elite skiers prefer the DS at an incline of ~7° (Dahl et al., 2017). The DP has been found to be skiers' preferred classical sub-technique on intermediate inclinations (2–4°) (Pellegrini et al., 2013; Andersson et al., 2016; Welde et al., 2017; Strøm Solli et al., 2020); for these inclinations, the DP is associated with a lower oxygen cost and higher GE than the DS (Hoffman et al., 1994; Pellegrini et al., 2013; Andersson et al., 2016; Dahl et al., 2017). Hence, a higher GE is related to reduced metabolic demand for a given skiing speed, which would be advantageous for performance when using the DP compared to using the DS on intermediate terrain.

The technique development in the DP has enabled this sub-technique to be used more extensively on a variety of inclines (Stöggl and Holmberg, 2016), and in the 15-km classical technique race of the 2016 Norwegian championship, the winner used the DP for propulsive force contribution throughout the race (Welde et al., 2017). On intermediate terrain in a 10 or 15 km race, analyses showed that faster skiers used the DP and DPK to a greater extent than slower skiers (Stöggl et al., 2018), and recently, it was found that elite male skiers used the DP 77% of their skiing time on inclines between 2 and 4° during a 15-km race (Strøm Solli et al., 2020). Furthermore, it was reported that elite male skiers improved their skiing performance by ~23 s in a 5-km race when they used the DP exclusively compared with when they skied with a free choice of sub-techniques within the classical technique (Stöggl et al., 2019). Hence, DP is a frequently used sub-technique among elite male skiers, and performance on flat terrain has been suggested to be more important for competitive success in elite male skiers than in elite female skiers (Stöggl et al., 2018).

There are two main phases in the DP: the repositioning phase and the poling phase. During the repositioning phase, the skier extends the joints that are flexed during the previous force transfer (i.e., ankle, knee, and hip joints) to reposition the body into an upright position with a simultaneous raising of the center of body mass (CoM). This increase in gravitational potential energy is thereafter transformed, through the poles, to kinetic energy for forward propulsion during the poling phase. During this phase, the force produced by skeletal muscles also contributes to propulsion.

The “old-fashioned” DP (DP_OLD_), used in the 1980s and 1990s, was characterized by pronounced trunk flexion and elbow extension at the later stage of the poling phase, where the skier's hands and pole handles pass below knee level with a simultaneous forward inclination of the poles to increase the horizontal propulsive force components (Smith, 2003). It has been suggested that trunk flexion not only lowers the arms and pole handles but also allows the shoulders and elbows to remain in their mid-range positions, where greater joint torque can be generated; this causes more poling force to be exerted in the poling-phase sequence in which the poles are effectively inclined (Smith, 2003). The DP started to evolve in the beginning of the 21st century and today “modern” DP (DP_MOD_) is the technique predominantly used by elite skiers (Pellegrini et al., 2018a). The DP_MOD_ is characterized by reduced angular joint movements accompanied by higher flexion velocities and greater poling forces (Holmberg et al., 2005), and compared to the characteristics of the DP_OLD_, this technique development leads to shorter poling times and thereby an improved ability to generate higher skiing speeds in flat terrain (Lindinger et al., 2009). As a result of the reduced joint flexion, compared to that of the DP_OLD_, the CoM is in a higher position when the repositioning phase is initiated, and reduced work against gravity is necessary to reposition the body before the subsequent poling phase.

Hence, from a biomechanical perspective the DP_OLD_ and DP_MOD_ differ, and it was previously reported that the CoM displacement within the DP_MOD_ explains differences in energy cost between groups with different levels of performance ability (Zoppirolli et al., 2015). Recently, it was shown that a pronounced trunk inclination was related to an increased energy cost during DP (Pellegrini et al., 2018b). They also proposed that, during the last three decades, the DP technique among elite skiers has evolved from a technique characterized by pronounced trunk flexion toward a technique with greater emphasis on shoulder motion during the propulsion phase (Pellegrini et al., 2018b). However, no previous study has investigated whether these biomechanical differences are reflected by physiological differences between techniques. The purpose of the study was to investigate whether there are energy-efficiency differences between the execution of the DP_OLD_ and DP_MOD_ at a submaximal work intensity among elite male cross-country skiers. It was hypothesized that execution of DP_OLD_ would be associated with higher mean oxygen uptake (VO_2_), respiratory exchange ratio (RER), metabolic rate (MR), and blood lactate concentration (BLa) as well as lower GE compared to the values observed when the DP_MOD_ was used.

## Materials and Methods

### Participants

Fifteen elite male cross-country skiers (age: 22 ± 4 years; stature: 183 ± 9 cm; body mass: 79.0 ± 9.9 kg) volunteered to participate in the study, and 10 of the skiers had competed in the World Ski Championships and/or the World Cup.

### Testing Procedures

Prior to the tests, the height of each participant was measured (Harpenden Stadiometer, Holtain Limited, Crymych, Great Britain). Thereafter, the total mass (*m*) of each participant and his equipment (i.e., roller skis, poles, ski boots, safety harness, heart-rate receiver, gloves, and clothes) were analyzed (Midrics 2, Sartorius AG, Goettingen, Germany). The participants' own poles were equipped with plastic tips (black plastic tip; LEKI Lenhart GmbH, Kirchheim, Germany) to allow skiers to achieve an adequate grip on the belt of the motor-driven treadmill (Saturn, 450/300 rs, h/p/cosmos sports & medical GmbH, Nussdorf-Traunstein, Germany) during the double-poling tests. The roller skis (Pro-Ski C2, Sterner Specialfabrik AB, Dala-Järna, Sweden) was provided by the laboratory, and the coefficient of rolling resistance of the roller skis (μ) was determined to be 0.022 by using the negative-inclination-equilibrium method previously described (Carlsson et al., 2016).

Before the warm-up, the participants were shown a video clip from the 50 km competition during the Olympics in Sarajevo 1984 (Gullemun, 2009), where the skiers used the DP_OLD_. In the video clip, the skier had great hip flexion where the trunk in the lower position was almost parallel to the surface. Another technique-specific characteristic for the DP_OLD_, which was shown in the video, was that the skier's arms were almost fully extended at the pole plant and the skiers' hands passed below the knees at the end of the propulsive phase. Moreover, to be familiar with the execution of the DP_OLD_, the participants tried the technique during the initial 4 min of the warm-up at a treadmill speed of 2.2 m/s (i.e., 8.0 km/h) and an inclination (α) of 2.5°. Throughout the familiarization period, the individuals conducting the test gave feedback on the skiers' execution based on the technique-specific characteristics to ensure that the participants were able to execute the DP_OLD_ properly. For the remaining 8 min of the warm-up, the treadmill speed was adjusted to find an adequate work intensity for each participant to use during the double-poling tests. The speed adjustments started from a precalculated treadmill speed, which was based on an approximation of an appropriate MR during the double-poling tests. The participants estimated their VO_2_max, based on previous test results, and 90% of this value was expected to indicate the peak aerobic power during DP (VO_2_peak) (Holmberg et al., 2007; Björklund et al., 2010; Skattebo et al., 2019). The VO_2_peak was then multiplied by a factor of 0.82, which was derived from unpublished data collected during a previous study (Carlsson et al., 2014), to establish an MR equivalent to a BLa of ~2.0 mmol/l. Based on the predetermined μ, *m*, α, and an estimated GE of 16.5% (Dahl et al., 2017), the treadmill speed corresponding to the approximated MR was calculated. Additionally, in the last 4 min of the warm-up, the participants used the diagonal-stride technique at a work intensity (~2.8 m/s, 5.0°) corresponding to the intensity of the subsequent double-poling tests, to minimize differences in BLa values prior to each specific test. Immediately after the warm-up, capillary blood samples were collected to determine the participants' BLa (Biosen 5140, EKF-diagnostic GmbH, Barleben, Germany) prior to the performance of the first double-poling test.

During the first test, the participants were instructed to use their ordinary double-poling technique (i.e., DP_MOD_), and 1 min after the warm-up, the 4-min test was initiated. The submaximal work intensity during the DP_MOD_ test was constant with an α of 2.5°, and the fixed treadmill speed (*v*) varied between 3.5 and 4.0 m/s (i.e., 12.6 and 14.3 km/h) depending on the physiological status of the participant. During the 1-min pause between tests, capillary blood samples were collected from the participants. Thereafter, the participants performed the 4-min test using the DP_OLD_ at the same individual submaximal work intensity. Throughout both double-poling tests, the skiers' expired air was continuously analyzed using a metabolic cart in mixing-chamber mode (Jaeger Oxycon Pro, Erich Jaeger Gmbh, Hoechberg, Germany) to continuously determine VO_2_, RER, ventilation (VE), and breathing frequency (BF).

The calculations of MR and GE were based on the VO_2_ (l/min) and the RER (l/l) during the last minute of each test. The MR (J/s) was determined using the formula (3.815 + 1.232 · RER) · VO_2_ · k_1_ (Lusk, 1928), where k_1_ was 69.73 and converted kcal/min to J/s. GE is the ratio of the mechanical work rate (MWR) to MR. Based on basic physics, the MWR (J/s) is the sum of the work against gravity and the work related to overcoming the rolling resistance of the roller skis; hence, the MWR was calculated in accordance with the formula *m* · *g* · sin α · *v* + *m* · *g* · cos α · μ · *v*, where *g* is the acceleration due to gravity.

**Figure 1:**
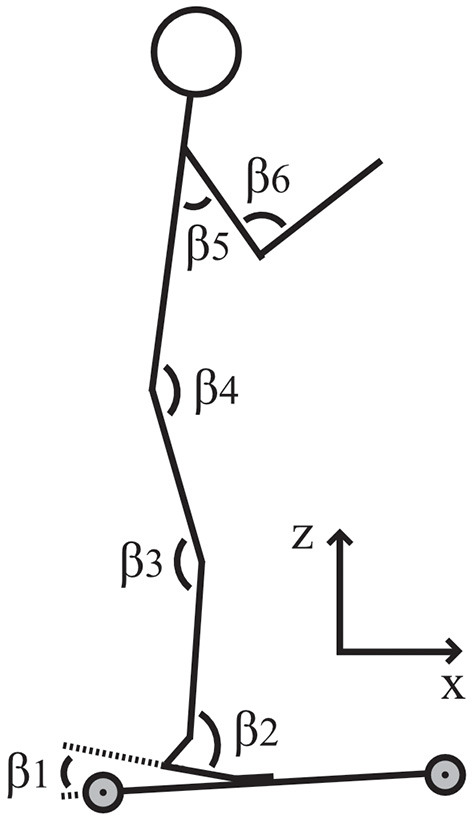
Analyzed joint angles of the skiers.

All double-poling tests were recorded using a video camera (Logitech Rally Camera, Logitech International S.A., Lausanne, Switzerland) positioned perpendicular to the skiing direction with a 3.4-m distance between the camera and the right side of the skiers' body. The video recordings were made to enable subsequent analyses of their sagittal joint angles at the highest and lowest positions during a DP cycle using a video analysis program (Live S, Dartfish SA, Fribourg, Switzerland). The analyzed angles for the two positions were β_1_, heel; β_2_, ankle; β_3_, knee; β_4_, hip; β_5_, shoulder; and β_6_, elbow (Figure 1). For each technique, the joint angles were analyzed for four double-poling cycles at 45 s, 35 s, 25 s, and 15 s before the end of the test. Moreover, the participants' technique-specific cycle rate (CR), and thereby the cycle length (CL), was determined by analyzing the last minute of the video recording.

To estimate the CoM for the two analyzed positions of each double-poling cycle, the length of each body segment, as a fraction of the stature, was determined using a previously published humanoid model (Winter, 2009). Based on the segments' lengths and the six joint angles, the x-coordinate and z-coordinate for each joint center were calculated. Thereafter, the segments' mass and its CoM were determined using a standard model (Robertson et al., 2014). The participants' vertical CoM in each position was calculated as the sum of each segment's mass multiplied by the z-coordinate of the CoM of the segment divided by the body mass. The vertical CoM displacement during each double-poling cycle was calculated as the difference in the vertical CoM between the highest and lowest positions.

### Statistics

The results for the biomechanical and physiological variables are presented as the means and standard deviations. The normality of the distributions of test variables was assessed by using the Shapiro–Wilk test. For each test variable, a 95% confidence interval (95% CI) was calculated for the difference between double-poling techniques. Hedges' g, with a correction for small sample size, was used to interpret the magnitude of the effect size (*ES*) and to enable more informative inferences to be made from the results. Interpretations of the size of the effect were as follows: 0.2 ≤ *ES* < 0.5 signified a small effect, 0.5 ≤ *ES* < 0.8 indicated a moderate effect, and *ES* ≥ 0.8 denoted a large effect (Cohen, 1988). All statistical analyses were assumed to be significant at an alpha level of 0.05. The statistical analyses were conducted using IBM SPSS Statistics software, Version 26 (IBM Corporation, Armonk, USA).

## Results

There were technique-specific differences in the joint angles between the DP_MOD_ and DP_OLD_ at the highest position for heel (*t* = 5.34; *P* < 0.001; 95% CI [5.48, 12.94]; *ES* = 1.37), ankle (*t* = 4.92; *P* < 0.001; 95% CI [5.09, 13.06]; *ES* = 1.19), knee (*t* = −4.73; *P* < 0.001; 95% CI [−5.10, −1.90]; *ES* = −0.78), shoulder (*t* = −11.34; *P* < 0.001; 95% CI [−36.82, −25.04]; *ES* = −3.45), and elbow (*t* = −10.59; *P* < 0.001; 95% CI [−43.86, −29.00]; *ES* = −2.84), whereas no difference was found in the measurements of hip (*t* = −0.57; *P* = 0.58; 95% CI [−2.74, 1.60]; *ES* = −0.086) (Table 1). For the lowest position, there were significant differences between the DP_MOD_ and DP_OLD_ in the skiers' joint angles ankle (*t* = −4.37; *P* < 0.001; 95% CI [−4.80, −1.63]; *ES* = −0.73), hip (*t* = 10.66; *P* < 0.001; 95% CI [19.88, 29.98]; *ES* = 3.55), shoulder (*t* = −8.50; *P* < 0.001; 95% CI [−33.41, −19.87]; *ES* = −2.98), and elbow (*t* = −6.43; *P* < 0.001; 95% CI [−32.45, −16.12]; *ES* = −1.82); however, there were no differences in the measurements of the skiers' heel (*t* = 0.00; *P* = 1.00; 95% CI [NaN]; *ES* = NaN) and knee (*t* = 0.00; *P* = 1.00; 95% CI [−3.39, 3.39]; *ES* = 0.00) (Table 1). The presented results related to the video analyses are based on 14 participants because the recording of one participant was corrupted.

**Table 1:** Skiers' joint angles in the highest and lowest position for each double-poling technique (mean ± standard deviation).

**Joint angle**	**DP_**MOD**_ (High)**	**DP_**MOD**_ (Low)**	**DP_**OLD**_ (High)**	**DP_**OLD**_ (Low)**
β_1_ (heel)	15 ± 5^***^	0 ± 0	6 ± 7	0 ± 0
β_2_ (ankle)	94 ± 7^***^	83 ± 5^***^	85 ± 8	87 ± 4
β_3_ (knee)	167 ± 4^***^	142 ± 6	170 ± 5	142 ± 7
β_4_ (hip)	161 ± 7	96 ± 8^***^	161 ± 6	71 ± 5
β_5_ (shoulder)	49 ± 9^***^	10 ± 10^***^	80 ± 9	36 ± 8
β_6_ (elbow)	89 ± 12^***^	108 ± 14^***^	126 ± 13	132 ± 12

DP_MOD_, modern double-poling technique; DP_OLD_, old-fashioned double-poling technique; High, highest position during the double-poling cycle; Low, lowest position during the double-poling cycle. Significant technique-specific differences between DP_MOD_ and DP_OLD_ at the highest position and lowest position are reported as ^*^P < 0.05, ^**^P < 0.01, and ^***^P < 0.001.

**Figure 2:**
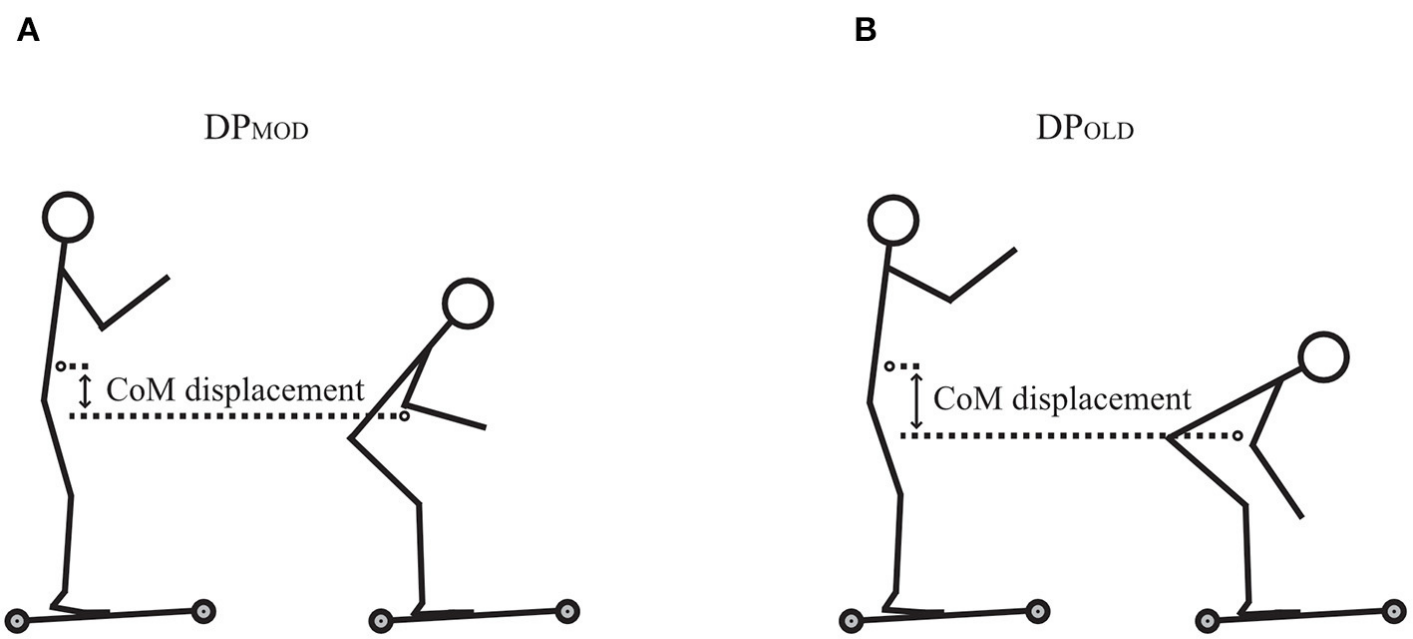
Skiers' mean vertical center of mass (CoM) displacement between the highest and lowest positions in the **(A)** DP_MOD_ and **(B)** DP_OLD_ tests.

Skiers' vertical CoM at the highest position when using the DP_MOD_ and DP_OLD_ were 1.12 ± 0.06 m and 1.11 ± 0.06 m, respectively (Figure 2). The skiers' corresponding CoMs for the lowest position were 0.94 ± 0.06 m for the DP_MOD_ and 0.83 ± 0.05 m for the DP_OLD_ (Figure 2). There was a significant difference in skiers' vertical CoM displacement between the DP_MOD_ and DP_OLD_ (0.19 ± 0.02 m vs. 0.28 ± 0.03 m) (*t* = −10.59; *P* < 0.001; 95% CI [−11.36, −7.51]; *ES* = −3.33).

Skiers' CRs during the DP_MOD_ and DP_OLD_ tests were 0.86 ± 0.06 Hz and 0.69 ± 0.06 Hz, respectively. Skiers' corresponding CLs were 4.36 ± 0.27 m for the DP_MOD_ test and 5.47 ± 0.45 m for the DP_OLD_ test. The CRs were significantly higher during the DP_MOD_ test (*t* = 9.97; *P* < 0.001; 95% CI [0.14, 0.21]; *ES* = 2.77), whereas the CLs were significantly greater during the DP_OLD_ test (*t* = −9.69; *P* < 0.001; 95% CI [−1.36, −0.86]; *ES* = −2.90). Skiers' total CoM displacement per minute from the lowest position to the highest position was 9.82 ± 1.20 m during the DP_MOD_ test and 11.73 ± 1.45 m during the DP_OLD_ test. The corresponding work related to lifting the CoM against gravity was 127 ± 24 J/s for the DP_MOD_ test and 151 ± 28 J/s for the DP_OLD_ test, where skiers performed significantly greater amount of work during the DP_OLD_ test (*t* = −5.47; *P* < 0.001; 95% CI [−2.07, −0.90]; *ES* = −0.91).

The results for the physiological variables collected during the last minute of each test as well as after the tests are presented in Table 2.

**Table 2:** Test results for the physiological variables (mean ± standard deviation).

**Variable**	**DP_**MOD**_**	**DP_**OLD**_**
VO_2_ (l/min)	3.41 ± 0.38	3.47 ± 0.45
VO_2_ (ml/min/kg)	43.4 ± 2.9	44.0 ± 3.2
RER (l/l)	0.95 ± 0.03^*^	0.93 ± 0.03
MR (J/s)	*1, 187*±130	*1, 200*±153
MWR (J/s)	199 ± 25	199 ± 25
GE (%)	16.8 ± 0.8	16.6 ± 0.8
BLa_pre_ (mmol/l)	2.0 ± 0.6^*^	2.4 ± 0.8
BLa_post_ (mmol/l)	2.4 ± 0.8	2.6 ± 0.9
BLa_diff_ (mmol/l)	0.4 ± 0.6	0.3 ± 0.6
VE (l/min)	86.5 ± 9.8^**^	92.5 ± 11.4
BF (breaths/s)	0.59 ± 0.15	0.63 ± 0.08

DP_MOD_, modern double-poling technique; DP_OLD_, old-fashioned double-poling technique; VO_2_, mean oxygen uptake; RER, respiratory exchange ratio; MR, metabolic rate; MWR, mechanical work rate; GE, gross efficiency; BLa_pre_, blood-lactate concentration pre-test; BLa_post_, blood-lactate concentration post-test; BLa_diff_, difference in blood-lactate concentration between post-test and pre-test; VE, ventilation; BF, breathing frequency. Significant differences between DP_MOD_ and DP_OLD_ are reported as ^*^P < 0.05, and ^**^P < 0.01.

There were no significant differences between measurements taken during the DP_MOD_ and DP_OLD_ tests for either VO_2_ (*t* = −1.37; *P* = 0.19; 95% CI [−0.14, 0.03]; *ES* = −0.14), MR (*t* = −1.01; *P* = 0.33; 95% CI [−43.17, 15.57]; *ES* = −0.09) or GE (*t* = 0.74; *P* = 0.47; 95% CI [−0.27, 0.56]; *ES* = 0.17) (Table 2). The MWR was the same in both test conditions (*t* = 0.00; *P* = 1.00; 95% CI [NaN]; *ES* = NaN), but the RER was higher during the DP_MOD_ test than during the DP_OLD_ test (*t* = 2.20; *P* = 0.045; 95% CI [0.0004, 0.03]; *ES* = 0.43) (Table 2). Skiers' BLa_post_ values were not significantly different (*t* = −1.70; *P* = 0.11; 95% CI [−0.57, 0.07]; *ES* = −0.30), although their BLa_pre_ values were higher before the DP_OLD_ test (*t* = −2.62; *P* = 0.020; 95% CI [−0.69, −0.07]; *ES* = −0.51). No significant difference in BLa_diff_ values was found between tests (*t* = 0.53; *P* = 0.060; 95% CI [−0.39, 0.64]; *ES* = 0.22). The VE was significantly lower during the DP_MOD_ than during the DP_OLD_ (*t* = −3.11; *P* = 0.0076; 95% CI [−10.08, −1.86]; *ES* = −0.55), but no difference between techniques was found for BF (*t* = −1.44; *P* = 0.17; 95% CI [−0.11, 0.02]; *ES* = −0.32).

## Discussion

The results presented herein show that DP_OLD_ and DP_MOD_ differ substantially from a biomechanical perspective, where many of the analyzed joint angles in the highest and lowest positions during the DP cycle were significantly different between the two techniques. These biomechanical differences resulted in a greater CoM displacement for each DP cycle and a significantly greater amount of work related to lifting the body mass against gravity per minute as well as a lower CR (i.e., greater CL) when skiers performed the DP_OLD_ than when skiers performed the DP_MOD_. Despite the technique-specific differences, the results of this study demonstrated that there were no substantial energy-efficiency differences between DP_OLD_ and DP_MOD_ at a submaximal work intensity, as indicated by the lack of significant between-test differences for VO_2_, MR, GE, and BLa_diff_ measurements.

The novel finding that DP_OLD_ is not associated with an increased energy expenditure compared to DP_MOD_ was somewhat unexpected. Based on the theoretically greater CoM displacement using DP_OLD_ and the technique development and refinement during the last decades, we hypothesized that execution of the DP_OLD_ would be more physically demanding than the DP_MOD_ and should therefore be related to a higher oxygen consumption and consequently a higher energy expenditure for the standardized submaximal work intensity. Based on the results, the hypothesis was rejected despite the pronounced biomechanical differences noted between the techniques, where the majority of the measured joint angles in the highest and lowest positions (9 out of 12) differed significantly between techniques.

To explain the non-significant difference in energy expenditure, it is necessary to compare the two techniques from an energetic perspective. During the roller-skiing tests, the energy demand related to work against gravity and the work related to overcoming the rolling resistance of the roller skis was equal for both tests. Moreover, while roller skiing on a treadmill, there is no energy expenditure related to air resistance. Therefore, the MWR was the same for both DP tests. However, it could be assumed that DP_OLD_ and DP_MOD_ differ biomechanically in three factors during the DP cycle: increase in potential energy, translational kinetic energy, and rotational kinetic energy.

There was a significant between-technique difference in the skiers' CoM displacement for each DP cycle (28 cm during DP_OLD_ vs. 19 cm during DP_MOD_). These results are in line with the previously reported displacements of 25–30 cm for the DP_OLD_ (Smith, 2003), and ~18–19 cm for elite male skiers using the DP_MOD_ while roller skiing at an inclination/speed combination similar to the combination used in the current study (Zoppirolli et al., 2015; Danielsen et al., 2019). The difference between techniques in terms of energy expenditure related to lifting the CoM against gravity is somewhat reduced because of the lower CR of DP_OLD_. In total, the increase in potential energy associated with CoM displacement is ~19% greater using DP_OLD_ (151 J/s during DP_OLD_ vs. 127 J/s during DP_MOD_). Consequently, the more pronounced trunk flexion using DP_OLD_, indicated by the smaller hip angle in the lowest position compared to that of DP_MOD_, is disadvantageous for DP_OLD_ from an energetic perspective. This is in line with a previous study that showed that the CoM displacement explained differences in energy cost between groups with different levels of performance ability (Zoppirolli et al., 2015).

The translational kinetic energy depends on the difference between the squared highest and lowest velocities during the stride cycle, where a higher CR is assumed to minimize power fluctuations and thereby reduce the energy demand related to this factor (Bergh, 1987). Hence, a large variation in intra-cycle speeds, between minimum speed and maximum speed within the DP cycle, results in a greater acceleration of body mass compared to a situation with lower intra-cycle speed variation. Acceleration of the body is costly from an energetic perspective and in line with this statement a significant correlation was found between an increase in intracycle variation in swimming speed and energy cost (Barbosa et al., 2005). Therefore, a greater CR would be preferable to minimize the energy expenditure associated with the translational kinetic energy. However, it has been shown that skiing with an imposed excessively high CR of 80 cycles/min is associated with a significantly lower GE compared to a CR of 40 cycles/min (Lindinger and Holmberg, 2011). The greater energy demand is to some extent related to the increased rotational kinetic energy due to higher angular velocities for body segments involved during the DP cycle. In the current study, the CR was higher when performing the DP_MOD_. However, the moment of inertia is probably higher for DP_OLD_, because of the greater joint angle in the elbow throughout the DP cycle. Therefore, a more comprehensive biomechanical analysis is necessary to determine which technique is more energy demanding from a rotational kinetic energy perspective.

The total energy expenditure linked to these three biomechanically related factors (i.e., increase in potential energy, translational kinetic energy, and rotational kinetic energy during the DP cycle) does not differ significantly between DP_MOD_ and DP_OLD_, and the advantages and disadvantages for each technique are outbalanced from an energetic perspective. As a consequence of the nonsignificant difference in MR between techniques and the higher CR for DP_MOD_, it could be concluded that the energy expenditure per DP cycle is higher for DP_OLD_. Therefore, the ratio between propulsive force impulse and energy expenditure is greater when using the DP_OLD_ than when executing the DP_MOD_. The greater hip flexion together with the hands passing below the knees results in a relatively large angular displacement of the ski poles at the later stage of the poling phase during DP_OLD_, which will thereby increase the poling force component in the direction of the track (Hoffman et al., 1990). Through the more effective positioning of the poles during the DP_OLD_, as much as 90% of the poling force contributes to propulsion (Smith, 2003). Together with the finding that the extensor muscles in the shoulder and elbow joints remain in their mid-range positions during the later stage of the poling phase (Smith, 2003), the more effective force contribution to forward propulsion when executing DP_OLD_ results in longer CL compared to the CL when using DP_MOD_.

The longer poling time and higher propulsive force impulse when performing the DP_OLD_ did not generate higher values of either BLa_diff_ or BLa_post_ than after the DP_MOD_ test. One possible explanation for the non-significant difference in BLa between techniques is the greater absolute recovery time during the repositioning phase when using DP_OLD_, which allows a better blood flow with oxygenated blood to the force-producing muscles. Even at moderate intensities during DP execution, force production by the arms is suggested to lead to mechanical hindrance of the oxygen supply, resulting in a lower oxygen extraction in the arms than in the legs (Stöggl et al., 2013). An impaired oxygen supply to the arm musculature implies that there is a higher reliance on glycolytic type II muscle fibers for force production; thus, there is increased lactate production (Ahlborg and Jensen-Urstad, 1991; van Hall, 2000). This reasoning is in line with a recent review in which it was suggested that a rapid force application requires a greater involvement of type II muscle fibers and that a DP strategy with longer poling time could thereby reduce energy expenditure (Zoppirolli et al., 2020b).

The RER was significantly higher during the DP_MOD_, which to some extent could reflect a more extensive use of type II muscle fibers. Another potential explanation for the reduced RER when performing the DP_OLD_ is related to the higher VE. It has previously been reported that ventilation and saturation are better during DP than during DS, because during execution of the DP technique the skier bends the upper body from an upright position to a nearly horizontal position by contraction of the abdominal muscles which in turn assists exhalation (Holmberg and Calbet, 2007). As indicated by the biomechanical differences between the DP_MOD_ and the DP_OLD_, the bending motion is more pronounced during DP_OLD_ contributing to higher tidal volume by a reduction of end-expiratory lung volume compared to that when executing the DP_MOD_. Another important factor for the ventilation difference between the two DP techniques is the better synchronization of respiratory and poling frequencies during DP_OLD_; the ratio between BF and CR is close to 1:1 when DP_OLD_ is used, whereas the corresponding ratio when using DP_MOD_ is ~1:1.5.

In total, the aerobic energy expenditure was equivalent for both techniques, as indicated by the lack of significant differences in VO_2_, MR, and GE. The GE of 16.8% in the DP_MOD_ test was in line with a previous study, which reported a mean GE value of ~16.7% for elite male skiers at a similar inclination/speed combination (Dahl et al., 2017). Previously, it has been suggested that individual differences in exercise efficiency are influenced by a weighted sum of physiological and biomechanical factors (Williams and Cavanagh, 1987). In line with this suggestion, exercise efficiency has been suggested to be determined by cardiorespiratory, metabolic, neuromuscular and biomechanical efficiencies (Barnes and Kilding, 2015). Additionally, for skiers of different levels of performance, one or several of these efficiencies could differ between groups, which ultimately results in a higher MR for a given MWR and thereby a lower GE for regional-level skiers.

Performance-level differences in GE have previously been found (Sandbakk et al., 2010; Ainegren et al., 2013), but in the current study, we investigated one group of elite male skiers who performed two different techniques. From the results, it was reasonable to assume that their cardiorespiratory, metabolic, and neuromuscular efficiencies did not differ between DP_OLD_ and DP_MOD_ execution. However, despite significant differences in joint angles and CoM displacement between techniques, GE values did not differ, which suggests that the generally accepted greater biomechanical efficiency for the DP_MOD_ is not correct, at least when DP at a submaximal work intensity. This suggestion is supported by results from computer simulations of skiing efficiency in the double-poling technique using a 3D full-body musculoskeletal simulation model (Holmberg et al., 2013), which found that the traditional technique (i.e., similar to the DP_OLD_) had a 0.4% higher efficiency than the modern technique.

However, when the DP speed reaches maximum or close to maximum, there is need for a high force impulse to achieve a long CL (Stöggl and Holmberg, 2011, 2016), which requires that the skiers use and master the execution of DP_MOD_ (Stöggl et al., 2011; Zoppirolli et al., 2017a,b). Recently, it was shown that better skiers had shorter duty cycles (% time of the poling phase within the poling cycle), as a result of a shorter poling phase and longer reposition phase compared to skiers with a lower level of performance (Zoppirolli et al., 2020a).

## Limitations

There are several limitations in this study. First, the participants did not use the DP_OLD_ in their daily training, which could be considered a limitation of the current study. However, despite their limited experience using DP_OLD_, there was no difference in energy efficiency between techniques. It could be speculated that a training period, where the skiers used DP_OLD_ in their daily training, would improve the execution of the technique and an improved biomechanical efficiency would thereby lead to a higher GE. This speculation needs to be confirmed or disconfirmed in future studies. Another limitation in the current study is the non-randomized test order in that all skiers started with the DP_MOD_ test; this approach was chosen because we wanted to avoid a potential negative physiological effect of DP_OLD_ on DP_MOD_. However, in light of the results that DP_OLD_ was not less energy efficient, randomization of the test order would have been possible without significant carry-over effects between tests. In fact, the chosen test procedure might have disfavored DP_OLD_ with a somewhat higher physiological stress at the beginning of the test. Another important issue to further investigate is the effect of prolonged exercise using either DP_MOD_ or DP_OLD_ on muscular fatigue and GE. Moreover, it would be of great importance to analyze the effect of a work intensity closer to the intensity during a race on metabolic stress for the two DP techniques. It would also be of interest to investigate whether the double-poling techniques differs in terms of physiological stress when skiing on snow. Potentially, the load profile on the skis differ between techniques and the grip vax might therefore influence skiing friction differently for DP_MOD_ and DP_OLD_. All these aspects should also be considered when investigating elite female skiers and skiers with different levels of performance ability.

## Data Availability Statement

The raw data supporting the conclusions of this article will be made available by the authors, without undue reservation.

## Ethics Statement

The studies involving human participants were reviewed and approved by the Swedish Ethical Review Authority, Lund, Sweden (Dnr 2020-00775). The patients/participants provided their written informed consent to participate in this study.

## Author Contributions

TC and MC formulated the first concepts for the study, conducted data analysis and statistical analysis, and wrote the first draft of the manuscript. TC, WF, LW, MS, and MC designed the experimental protocol. LW and WF conducted the experiments. All authors read and approved the final manuscript.

## Conflict of Interest

The authors declare that the research was conducted in the absence of any commercial or financial relationships that could be construed as a potential conflict of interest.

## Publisher's Note

All claims expressed in this article are solely those of the authors and do not necessarily represent those of their affiliated organizations, or those of the publisher, the editors and the reviewers. Any product that may be evaluated in this article, or claim that may be made by its manufacturer, is not guaranteed or endorsed by the publisher.
